# The Notch pathway in ovarian carcinomas and adenomas

**DOI:** 10.1038/sj.bjc.6602719

**Published:** 2005-08-23

**Authors:** O Hopfer, D Zwahlen, M F Fey, S Aebi

**Affiliations:** 1 Department of Clinical Research, University of Bern, Bern, Switzerland; 2 Department of Hematology and Oncology, Charité, Campus Benjamin Franklin-Klinikum, University Medicine Berlin, Berlin, Germany; 3 Department of Medical Oncology, University Hospital, Bern, Switzerland

**Keywords:** ovarian adenoma, ovarian carcinoma, Notch

## Abstract

Elements of the Notch pathway regulate differentiation; we investigated the expression of such elements in epithelial ovarian tumours. A total of 32 ovarian tumour samples (17 adenocarcinomas, three borderline tumours, 12 adenomas), two human ovarian cancer (A2780, OVCAR3), and one ovarian surface (IOSE 144) cell lines were analysed. The expression of Notch pathway elements was assessed by RT–PCR, real-time PCR (Notch 1), and by immunoblots (Notch 1 extracellular domain (EC), HES1). The proliferation and colony formation of A2780 cells were measured after stable transfection with activated Notch 1 (intracellular domain). *Jagged 2*, *Delta-like-1*, *Manic Fringe*, and *TSL1* were expressed more frequently in adenocarcinomas whereas *Deltex*, *Mastermind*, and *Radical Fringe* were more frequent in adenomas. Quantitative PCR revealed decreased Notch 1 mRNA in ovarian adenocarcinomas compared with adenomas. The expression of Notch 1-EC protein was similar in benign and malignant tumours. HES1 protein was strongly expressed in 18/19 ovarian cancers and borderline tumours but not in adenomas. Transfecting A2780 cells with active Notch 1-IC resulted in a proliferative and colony formation advantage compared to mock transfected cells. Thus, Notch pathway elements are expressed in ovarian epithelial tumours and some of them are differentially expressed between adenomas and carcinomas. The Notch pathway could be a target for the development of therapies for ovarian cancer.

The cellular origin, histology, and biological properties of ovarian neoplasms is heterogeneous and complex. About 90% of ovarian tumours are adenomas and adenocarcinomas derived from ovarian surface epithelium (OSE) of coelomic origin. Currently, ovarian adenocarcinomas are thought to arise from OSE inclusion cysts that form after OSE injury such as ovulation. The impaired ability of inclusion cyst cells to acquire a mesenchymal phenotype and the appearance of a gland-like differentiation of the otherwise more pluripotent mesodermal precursors resembling OSE cells is a hallmark of carcinogenesis (Scully, 1995). The molecular pathogenesis of ovarian adenocarcinomas is unknown although some mechanisms have been elucidated recently. Loss of function mutations of tumour suppressor genes have been described such as p53 in late stage tumours (Kupryjanczyk *et al*, 1993), PTEN in endometroid ovarian carcinomas (Obata *et al*, 1998), BRCA1 and BRCA2 in familial ovarian cancers (Ford *et al*, 1998; Kote-Jarai and Eeles, 1999). Overexpression or amplification of oncogenes have been described for PI3K (Philp *et al*, 2001) and its downstream effector AKT2 (Cheng *et al*, 1992), EGF-R (Kohler *et al*, 1989), cMyc (Tashiro *et al*, 1992), K-ras (Enomoto *et al*, 1991) and c-erbB (HER2/neu) (Slamon *et al*, 1989). In a recent study we demonstrated that *p73*, a member of the *p53* family, is expressed in ovarian adenocarcinoma but rarely in adenoma (Zwahlen *et al*, 2000). P73 and p63 increase the expression of the Notch ligands Jagged 1 and 2 (Sasaki *et al*, 2002) leading to increased expression of the Notch target HES1 in coculture assays. It appears likely that deranged proliferation and cell fate determination processes are of particular importance in the development of ovarian cancer. The Notch pathway with its family of four mammalian Notch receptors and their numerous ligands of the Delta and the Jagged/Serrate group is playing an important role in cell fate determination and organogenesis during the embryonic development and in haematopoiesis (Artavanis-Tsakonas *et al*, 1999; Miele and Osborne, 1999; Milner and Bigas, 1999). Figure 1 outlines the Notch signalling network: the Notch genes encode transmembrane receptor proteins of about 300 kDa that undergo post-translational modification by intramolecular cleavage and heterodimer formation. Upon interaction with a ligand, a complex cleavage process of the Notch receptor is initiated, resulting in the release of the intracellular c-terminal domain (approximately 110 kDa). The activating ligands, including Jagged 1, Jagged 2 (JAG 1 and JAG 2), Delta-like-1, -3 and -4 (DLL1, DLL3 and DLL4), are mainly expressed on the surface of neighbouring cells. The intracellular Notch domain then translocates to the nucleus to interact with the transcriptional repressor CBFl/RBP-Jk (Hsieh *et al*, 1996), turning it into a transcriptional activator of numerous target genes such as the *HES1* gene, a member of the basic helix–loop–helix family of transcription factors (Bailey and Posakony, 1995; Davis and Turner, 2001). In developmental processes, Notch signalling often results in the preservation of a small subset of proliferating undifferentiated and multipotent progenitor cells. However, the net effect of Notch signalling tends to be highly dependent on the cellular context. In a small subset of human acute lymphoblastic T-cell leukaemia a constitutively active Notch 1 mutant functions as an oncogene (Ellisen *et al*, 1991). Conversely activated Notch 1 and Notch 2 have been shown to result in a growth arrest of small cell lung cancer cells, where it seems to act as a tumour suppressor gene (Sriuranpong *et al*, 2001). Furthermore, Notch 1 has been shown to function as a tumour suppressor gene in mouse skin (Nicolas *et al*, 2003). In the present study, we show that many elements of the Notch pathway are expressed in epithelial ovarian tumours with carcinomas having higher HES1 protein expression levels than adenomas indicating a stronger Notch pathway activation. Furthermore, we demonstrate a proliferative and survival advantage of stably Notch 1-IC (intracellular domain) transfected A2780 ovarian carcinoma cells. We propose that the Notch pathway could be a target for the development of novel therapies for ovarian cancer.

## MATERIAL AND METHODS

### Patient characteristics and tumour specimens

Tumour samples were obtained from 32 patients who underwent surgical resection of an ovarian tumour at the university hospitals of Berne and Zurich, Switzerland. Sample collection was in accordance with Swiss law and approved by the local institutional review board. At the time of surgery no patient had received chemotherapy. The tissues for the present study were selected by the surgeons who were careful to submit large (>1 cm) pieces of tissue that were macroscopically tumourous. Adjacent tissues were used for the pathological diagnosis. The age range of the patients was 40–83 years (median 61 years). Tumours were classified in accordance with the WHO standard criteria (Tavassoli and Devilee, 2003) as ovarian epithelial adenocarcinomas (17 cases), epithelial ovarian cancers of low malignant potential (three cases) and ovarian adenomas (12 cases). Tumour samples were snap frozen in liquid nitrogen in the operating theatre immediately after surgical removal.

### Cell lines and culture conditions

Human ovarian adenocarcinoma cell lines A2780 and 2008 (kindly provided by Dr S Howell, University of California San Diego, La Jolla, CA, USA) were maintained in RPMI-1640 medium (Sigma, St Louis, MO, USA) supplemented with 10% heat-inactivated fetal calf serum (FCS, Sigma), the human ovarian adenocarcinoma cell line OVCAR-3 (ATCC, Rockville, MD, USA) was maintained in DMEM (Sigma) supplemented with 10% heat-inactivated FCS. The human Hodgkin's lymphoma cell line L540 (DSMZ, Braunschweig, Germany) was maintained in RPMI-1640 medium supplemented with 10% heat-inactivated FCS, and the human ovarian surface epithelial cell line IOSE-144 (kindly provided by Dr N Auersperg, University of Vancouver, Canada) was maintained in MCDB105 with L-glutamine (Sigma) and 25 mM HEPES+Medium 199 with Earle's salt and L-glutamine (Sigma) (1 : 1) supplemented with 5% heat-inactivated FCS and 50 *μ*g ml^−1^ gentamicin (Sigma).

### RNA extraction and RT–PCR

After homogenisation of tumours using a tissue homogeniser (Polytron, Kinematica, Littau, Switzerland), total RNA was extracted using the RNeasy/QIAamp Kit (Quiagen, Basel, Switzerland). Contaminating genomic DNA was removed by DNase I treatment according to the manufacturer's instructions (RNase-Free DNase Set, Quiagen). Reversed transcription was performed using 1 *μ*g total RNA, 0.8 *μ*g primer oligo dT15 primers (Roche, Rotkreuz, Switzerland), 1.3 *μ*l PCR Nucleotide Mix, 10 mM deoxynucleotide triphosphate (Roche), 28 U rRNasin (Promega, Wallisellen, Switzerland), 5 *μ*l M-MLV 5 × buffer (Promega), 200 U M-MLV reverse transcriptase (Promega) in a final volume of 25 *μ*l. PCR was carried out at least as duplicates as follows: 5 *μ*l cDNA as template were added to a mastermix made of 3 *μ*l 10 × PCR buffer+Mg (Roche), 3 *μ*l glycerol, 3 *μ*l PCR nucleotide mix, 1 mM deoxynucleotide triphosphate (Roche), 0.8 U Taq DNA polymerase (Roche), sense and antisense primers with a final concentration of 266 nM. Thermocycler conditions were as described elsewhere (Zwahlen *et al*, 2000). The annealing temperature was optimised for each primer pair and 35–37 PCR cycles were applied. The following *human glyceraldehyde-3-phosphate dehydrogenase (GAPDH)* primers were used for quality control of RT-PCR for each cDNA generated, sense: 5-GAGCTGAACGGGAAGCTCACTGG-3; antisense: 5-CAACTGTGAGGAGGGGAGATTCAG-3. PCR products were separated on a 1% agarose gel containing ethidium bromide. The primers used for the detection of cDNA of Notch pathway genes are shown in Table 1.

### Real-time quantitative RT–PCR for hNotch 1

TaqMan real-time RT–PCR (TaqMan PCR detector 7700, Perkin-Elmer Applied BioSystems, Rotkreuz, Switzerland) was used for relative quantification of hNotch 1 (Gene Bank accession no. AF308602) gene expression in human ovarian tumour samples and cell lines as described previously (Leonard *et al*, 2000). The preparation of cDNA was carried out as described above. Dual labelled (FAM/TAMRA) gene-specific probes and TaqMan Universal PCR Master Mix (Perkin-Elmer Applied BioSystems, Rotkreuz, Switzerland) were used. Sequences of *hNotch 1* specific oligonucleotides were as follows, probe: 5′-CCGCTCTGCAGCCGGGACA-3′; forward primer: 5′-CACTGTGGGCGGGTCC-3′; reverse primer, 5′-GTTGTATTGGTTCGGCACCAT-3′ (Shou *et al*, 2001). *Notch 1* gene expression was normalised to the expression level of the housekeeping gene *porphobilinogen deaminase* (PBGD, Gene Bank accession no. Ml8799). Sequences for PBGD-specific oligonucleotides were as follows, probe: 5′-TGCGGCTGCAACGGCGGAAGAAA-3′; forward primer: 5′-GGAGCCATGTCTGGTAACGGCA-3′; reverse primer: 5′-GGTACCCACGCGAATCACTCTCA-3′. Primers were used at a final concentration of 300 *μ*M; the final probe concentration used was 150 *μ*M. In total, 2.5 *μ*l cDNA and 12.5 *μ*l TaqMan Universal PCR Master Mix were added to a final reaction volume of 25 *μ*l. A total of 45 TaqMan PCR-cycles were run under standard conditions.

### Western blot analysis

**Figure 2:**
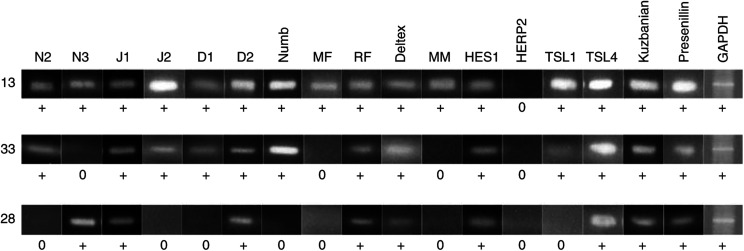
RT–PCR results of representative examples (13=ovarian adenocarcinoma, 33=borderline tumour, 28=ovarian adenoma) for Notch pathway elements. 0, no signal detected. +, mRNA detected by RT–PCR. The results of duplicate experiments were identical.

Cell extracts were prepared as described (Zwahlen *et al*, 2000). Briefly, after tissue homogenisation and sonication, cells were lysed in RIPA buffer (150 mM NaCl, 1.0% Nonidet P-40, 0.5% sodium desoxycholate, 0.1% sodium dodecyl sulfate, 50 mM Tris, pH 8.0) on ice for 30 min, followed by centrifugation for 20 min at 4°C. Quantification was done by Bradford assay (Bio-Rad, Glattbrugg, Switzerland). In all, 50 *μ*g total cellular protein per lane were size fractioned on a 7% tris-acetate gel (Invitrogen, Paisley, UK) for Notch 1 detection or on a 4–12% tris-glycine gradient gel (Invitrogen) for HES1 detection and blotted onto nitrocellulose (Protan; Schleicher and Schuell, Kassel, Germany). Equal loading and transfer efficiency was visually checked by Ponceau staining. Membranes were blocked for 3 h at room temperature with 5% w/v nonfat dry milk/TBS and Tween-20 (0.05% w/v). Membranes were incubated with rabbit polyclonal anti-Notch 1 antibodies (H-131, Santa Cruz, Nunningen, Switzerland) for the detection of the extracellular domain of Notch 1. For the detection of HES1, membranes were incubated with a rabbit polyclonal anti-HESl antibody (kindly provided by Dr Sudo, Toray Industries, Kanagawa, Japan). Detection of primary antibody binding was done with donkey antirabbit horseradish peroxidase conjugated antibodies, followed by enhanced chemiluminescence detection (all from Amersham, Zurich, Switzerland). Equal loading was assessed visually by detection of actin with a rabbit polyclonal antibody (A2066, Sigma) after stripping and reblocking of the membranes. The Hodgkin's Lymphoma cell line L540 served as a positive control for Notch 1 extracellular domain expression (Jundt *et al*, 2002). HES1 protein was *in vitro* synthesised by the TNT T7 Quick Coupled Transcription/Translation System (Promega) from the mHESl cDNA in pBluescript SK+ (kindly provided by Dr R Kageyama, Kyoto University, Japan). Each experiment was repeated at least twice, and the strenght of expression was scored visually by assigning one of four scores (strong, intermediate, weak, and absent; cf, Figure 2).

### Transfection and colony forming assay+proliferation assay

The ovarian adenocarcinoma cell line A2780 was transfected with *hNotch 1-IC-HA* (pCDNA3 Invitrogen) as previously described (Chen *et al*, 1997). Empty vector transfected cells were used as controls. Transfection was done by electroporation, applying 5 *μ*g plasmid DNA per 1 million cells. Selection was performed with G418 at 600 *μ*g ml^−1^. The resultant G418 resistant clones were pooled and used directly for colony forming and proliferation assays. The latter was carried out by the XTT method according to the manufacturer's instructions (Biological Industries, Beit Haemek, Israel). In total, 1000 cells were seeded in 9 cm wells in selection medium as indicated above. XTT assay was performed on days 4, 7, 8, 10 and 12. For the colony forming assays 300 cells of a single cell suspension were seeded in 10 cm Petri dishes and counted 7 days after when the colonies were visible by eye. All experiments were performed in triplicate.

### Dual luciferase-assay

Notch pathway activation of the stably *hNotch 1-IC-HA* transfected A2780 was tested by transient transfection of 1 million cells from pooled G418-resistant clones, using electroporation, with 5 *μ*g of a reporter plasmid containing the Notch/RBP-J*κ*-responsive region of the Hairy and Enhancer of Split homologue-1 (HES1) promoter as described previously (Rangarajan *et al*, 2001). Empty vector transfected cells served as controls. In all cases 100 ng of a renilla luciferase expression plasmid (pRL-CMV, Promega) were cotransfected. The Dual-Luciferase reporter assay (Promega) was performed according to the manufacturer's specifications 48 and 72 h after transfection.

## RESULTS

### RT–PCR analysis of the expression pattern of elements of the notch pathway in ovarian tumours

To test the hypothesis that the Notch pathway could be involved in the pathogenesis of ovarian carcinomas we studied the mRNA expression pattern of some of its elements by RT–PCR in 32 ovarian tumour samples (17 ovarian adenocarcinomas, three ovarian cancers of low malignant potential, 12 ovarian adenomas), three ovarian cancer cell lines (A2780, OVCAR-3, 2008), and one human ovarian surface epithelial cell lines (IOSE-144). *GAPDH* was detected in all samples, indicating similar quality of extracted mRNA. The results are summarised in Table 2 and representative samples of RT–PCR products are shown in Figure 2. *Notch 1* (see below), *Jagged 1*, *Delta-like-2/4*, *HES1* and *Presenillin* expression was detectable in all ovarian adenocarcinomas and ovarian adenomas (for Notch 1 only real-time RT–PCR data are shown). In contrast, ovarian adenocarinomas were more frequently positive than adenomas for the Notch ligands *Jagged 2* (*P*=0.04, Fisher's exact test, two-sided probabilities are shown without adjustment for multiple testing) and *Delta-like-1* (*P*=0.056), for the modulator of ligand specificity *Manic Fringe* (*P*=0.0007) and for the downstream transcription factor *Transducin-like Enhancer of split-1* (*TSL1*; *P*=0.01). On the other hand, slightly higher frequencies of expression were found in adenomas for Deltex and Mastermind, and Radical Fringe.

The expression pattern of elements of the Notch pathway in ovarian cancer cell lines was similar to ovarian adenocarcinomas with the exception of *Jagged 2*, *Delta-like-1*, *Delta-like-2* and *TSL4* that were expressed at a lower frequency.

### Real-time quantitative RT–PCR for *hNotch 1*

**Figure 3:**
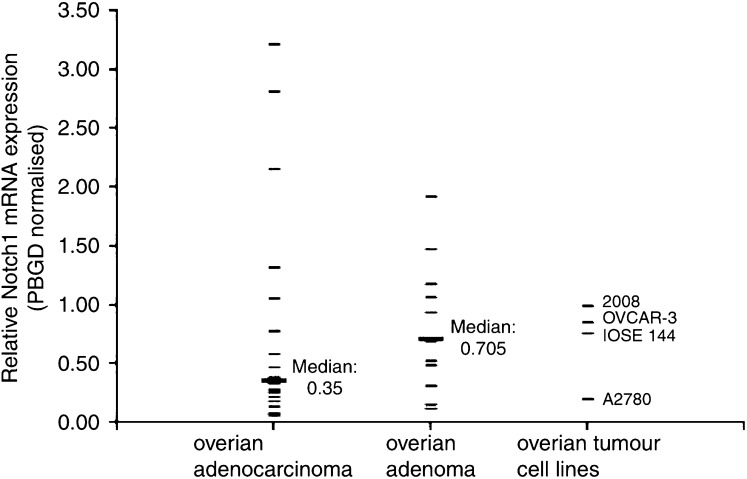
Notch 1 mRNA real-time PCR, normalised for PBGD (arithmetic means of triplicate measurements).

Altered *Notch 1* expression has been found in certain haematologic malignancies and solid tumours. To study the *Notch 1* mRNA expression level we used TaqMan real-time quantitative RT–PCR for the screening of human ovarian tumours and cell lines. Primers and probes were selected for the intracellular domain of *Notch 1*. We did not detect overexpression of *Notch 1* mRNA in ovarian adenocarcinomas (median expression level relative to PBGD : 0.35), but a tendency towards decreased *Notch 1* expression in comparison to ovarian adenomas (median : 0.705; Figure 3).

### Expression pattern of Notch 1 and HES1 protein

**Figure 4:**
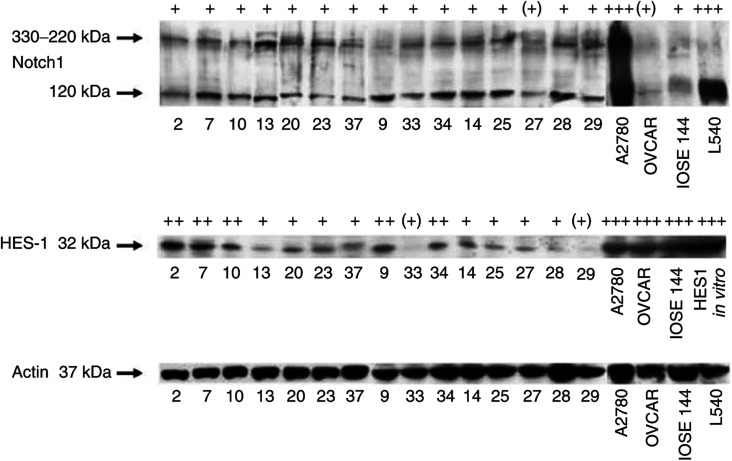
Western blots for Notch 1 (full-length and extracellular domain (EC)) and HES-1 in ovarian adenocarcinomas, borderline tumours, adenomas and cell lines (ovarian adenocarcinoma A2780 and OVCAR-3, ovarian surface epithelium IOSE-144 and Hodgkin's lymphoma L540).

We further investigated the protein expression pattern of full length Notch 1 (330–220 kDa), the Notch 1 extracellular domain (Notch 1-EC, 120 kDa) and of the downstream transcription factor HES1 (32 kDa) in 28 of the 32 ovarian tumours (16 ovarian carcinomas, three borderline tumours, nine ovarian adenomas), in three human ovarian cancer cell lines and one ovarian surface epithelium cell line. In four ovarian tumours the amount of extracted protein was insufficient for immunoblotting. The Hodgkin's lymphoma cell line L540 served as positive control for Notch 1-EC expression and *in vitro* translated mHESl cDNA for HES1 protein expression. Results are summarised in Table 3 and representative samples are depicted in Figure 4. Similar amounts of *β*-actin protein expression were detected in all tumour and cell line specimens. Notch 1 and Notch 1-EC protein was expressed in all ovarian cancers, borderline tumours and ovarian adenomas at similar levels. In ovarian cancer cell lines the expression of Notch was a strong expression of Notch 1-EC in A2780, an intermediate expression in OVCAR-3, but a lower expression level in IOSE-144.

The expression level of HES 1 protein on the other hand trended to be higher in the malignant than in the benign tumours: in invasive adenocarcinomas and borderline tumours, the expression was intermediate in 11 out of 19 and strong in seven out of 19 samples, whereas in ovarian adenomas five out of nine specimens showed intermediate and four out of nine weak or absent expression. However, in all ovarian adenocarcinoma and surface epithelium cell lines the HESl protein expression was very high at similar levels.

### Stable transfection of Notch 1-IC in A2780 ovarian adenocarcinoma cell line

**Figure 5:**
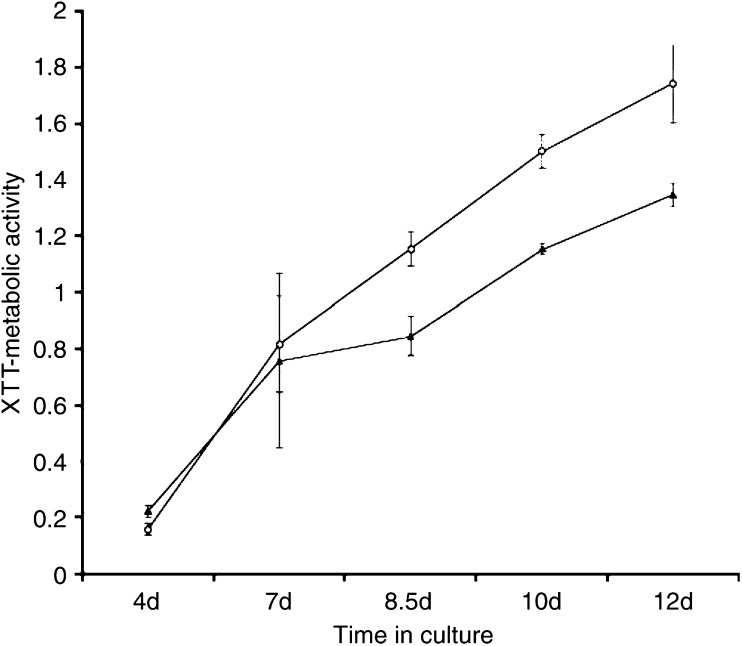
XTT-assay of A2780 human ovarian adenocarcinoma cells, stable transfections with *hNotch 1-IC-HA* (white circles) *vs* empty vector (black triangles) transfected.

The observation of a consistent expression of Notch 1 protein in ovarian adenocarcinomas and adenomas does not prove that Notch 1 signalling is actually active. The expression of HESl could be induced by other upstream factors such as Notch 2 or possibly Sonic Hedgehog (Shh) (Solecki *et al*, 2001). Therefore, we investigated the biological relevance of activated Notch 1 by stably transfecting the cell line A2780 with *Notch 1-IC*, the functionally active intracellular domain of the Notch 1 heterodimer, in order to increase the degree of Notch pathway activation. The biological activity of transfected *Notch 1-IC* was confirmed by a dual luciferase reporter-plasmid assay. The normalised luciferase activity was 127% higher in *hNotch 1-IC-HA* stably transfected A2780 than in empty vector controls 48 h after transient transfection and 72% after 72 h. When compared to the wild type, the *hNotch 1-IC-HA* transfected A2780 cells derived from pooled clones showed a proliferative advantage from day 7 onwards after cells reached confluence (Figure 5). The metabolic activity measured by the XTT-assay, mirroring cell mass, was on average 32±7.5% higher in the Notch 1-IC stably transfected A2780 cells compared to mock controls from day 7 on over the observation period. To test if the survival potential of A2780 was changed due to an increased Notch pathway activation, we carried out colony-forming assays. After 7 days approximately 28±12% more colonies formed from single cells of stably *hNotch 1-IC-HA* transfected cells compared to mock transfected controls.

## DISCUSSION

In the present study, we have shown differences in the mRNA and protein expression profile for certain elements of the notch pathway (receptors, ligands, modulators and downstream signalling factors) between ovarian adenocarcinomas, borderline tumours and ovarian adenomas: We could show a higher HESl protein expression in ovarian cancers and borderline tumours in comparison to adenomas. Transfection experiments with activated Notch 1 intracellular domain in A2780 ovarian adenocarcinoma cells indicated a proliferative advantage due to Notch 1 that is consistent with an impaired contact inhibition at confluence. The growth advantage of *Notch 1-IC* transfected A2780 cells in comparison to empty vector controls was also evident by their enhanced ability to form colonies from single cells on plastic surfaces (colony-forming units) (Bashey *et al*, 1992; Yu *et al*, 1999). The magnitude of *in vitro* growth effects was modest, but similar to recent observations with *c-Myc* (Pelengaris *et al*, 2002), the contribution to *in vivo* tumour formation could be significant. Altered Notch expression has already been observed in cervical carcinomas (Zagouras *et al*, 1995), lung cancer (Dang *et al*, 2003), pancreatic cancer (Miyamoto *et al*, 2003), breast cancer (Weijzen *et al*, 2002), and endometrium carcinomas (Suzuki *et al*, 2000). In early hematopoietic stem cells Notch-signalling stops differentiation and maintains a pool of proliferating pluripotent cells (Milner and Bigas, 1999; Ohishi *et al*, 2002; Kunisato *et al*, 2003). Of interest, the OSE is closer in its differentiation state to the pluripotent mesodermal embryonic precursor cells than other epithelial derivates (Scully, 1995). Although overexpression of *Notch 1* mRNA or protein has not been found in ovarian tumours, our data point to a possible role of the Notch pathway in ovarian cancer formation: our transfection experiments indicate, that activation of the Notch pathway could contribute to the malignant phenotype by maintaining proliferative activity against growth control signals and by promoting cell survival. Expression array data have revealed that at least two members of the Notch-signalling network may be involved in the ovarian carcinogenesis: *Notch 3* was at least three-fold overexpressed over normal ovarian surface epithelial cells (Lu *et al*, 2004) and a similar observation was reported for *Jagged 2* (Euer *et al*, 2005). These studies did not identify the same genes as overexpressed in ovarian cancer although they used similar methods; thus, these finding as well as our present study should be considered preliminary. How in detail the Notch pathway exerts its oncogenic function has not been fully elucidated. A recent study showed that CBF1, the first major downstream signalling factor of the Notch pathway, when activated, interacts with the Cyclin Dl promotor, resulting in increased CD1 activity and Gl/S-phase transition (Ronchini and Capobianco, 2001). Furthermore Notch-IC activated CBF1 induces the transcription of NF*κ*B(Oswald *et al*, 1998).

Besides it has been shown that NF*κ*B controls the expression of the Notch ligand Jagged 1 in a positive feedback loop (Bash *et al*, 1999). It is likely, however, that Notch alone is not capable of transforming cells but requires the functional collaboration of other oncogenes, namely from the RAS (IRK, MAP kinase, PI3 kinase) (Fitzgerald *et al*, 2000; Weijzen *et al*, 2002) and c-Myc pathways (Rao and Kadesch, 2003). PI3K (Philp *et al*, 2001) and c-Myc (Tashiro *et al*, 1992) overexpression have been reported for ovarian adenocarcinomas.

Several questions about the function of Notch in ovarian tumours still remain to be answered. The relevance of other receptors of the Notch family has not been elucidated yet. Likewise, it is not known what regulates the degree of Notch pathway activation in ovarian tumours. The expression pattern of Notch ligands might play a crucial role though. Interestingly our preliminary data point to a robust protein expression of Jagged 1 in ovarian tumours. If relevant cell–cell interactions are homotypic (between tumour cells themselves) or heterotypic (between tumour cells and other cells e.g. stroma cells) remains to be determined.

In summary, the Notch-signalling pathway is operative in ovarian cancer, and we propose a positive relationship between Notch pathway activation as indicated by HES1 protein expression levels and tumour growth in ovarian cancer. This hypothesis will need to be addressed in further studies. Moreover, elements of the Notch pathway could be worthwhile targets for future therapies of ovarian cancer (Jang *et al*, 2000; Nickoloff *et al*, 2003) such as siRNA and gamma-secretase inhibitors (Elbashir *et al*, 2001; Kimberly *et al*, 2003; Micchelli *et al*, 2003).

## Figures and Tables

**Figure 1:**
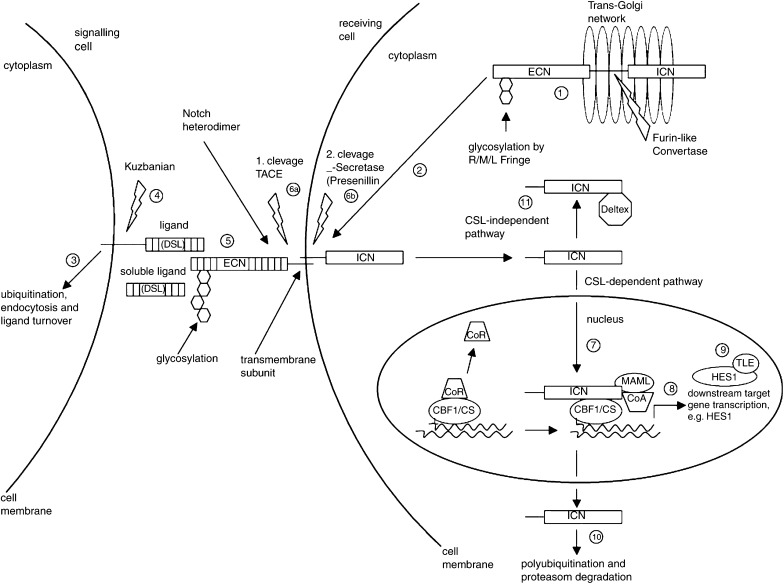
Notch signal transduction elements: (1) Post-translational modification of precursor Notch-protein includes cleavage by a Furin-like convertase and glycosylation by members of the Fringe family (Radical (R), Manic (M), Lunatic (L)) in the trans-Golgi. (2) Adherence of Notch extracellular domain (ECN) with Notch intracellular domain (ICN) results in mature Notch heterodimers that are transferred to the cell membrane. Receptor interaction with ligands of the DSL-family (Delta, Serrate, Lag3) on neighbouring cells is modulated by Fringe modification of ECN. (3) E3 ubiquitin ligases (Mindbomb and Neutralised) control the turnover of DSL-ligands on signalling cells. (4) Ligand availability is further regulated by the metalloprotease Kuzbanian, shedding the extracellular domain of the transmembrane ligand protein. (5) Successful interaction of extracellular ligand regions with EGF-like repeats of ECN lead to (6) successive cleavage of Notch transmembrane domain by the disintegrin-metalloprotease tumour necrosis factor-*α*-converting enzyme (TACE) and the *γ*-secretase Presenillin. (7) ICN is released and translocates to the nucleus where it interacts with CSL1, replacing CSL-repressors (CoR) and forming a transcription complex with Mastermind-like factors (MAML) and transcriptional coactivators (CoA). (8) This transcriptional complex activates downstream target genes, including members of the Hairy enhancer of split (HES) family of transcriptional repressors (bHLH). (9) These HES proteins excert their function together with the Transducin-like enhancer of split (TSL) family of corepressors. Other target genes are tissue specific. (10) The half-life of ICN is regulated by E3 ligases of the Sel-10 family before proteasome degradation. (11) A CSL-independent Notch pathway involves the interaction of ICN with deltex, a cytosolic protein and positive regulator of Notch signalling. Adopted from Weng and Aster (2004).

**Table 1:** RT-PCR primers for detection of Notch pathway genes

**Gene**	**Gene Bank accession number**	**Annealing temperature (°C)**	**Forward primer**	**Reverse primer**	**PCR product (bp)**
*Notch 2*	XM_016986	58	5-gatgccacctgaacaactgc-3	5-tgacaacagcaacagcaagg-3	407
*Notch 3*	XM_009303	58	5-ttgctgctggtcattctcg-3	5-tcctcttcagttggcattgg-3	476
*Jagged 1*	NM_000214	58	5-agcgacctgtgtggatgag-3	5-ggctggagactggaagacc-3	333
*Jagged 2*	NM_002226	58	5-tctctgtgaggtggatgtcg-3	5-cagtcgtcaatgttctcatgg-3	327
*Delta-like-1*	NM_003836	58	5-ccacgcagatcaagaacacc-3	5-ttgctatgacgcactcatcc-3	336
*Delta-like-2/4*	AB036931/AB043894	60	5-ccaactatgcttgtgaatgtcc-3	5-tgtggagaggtcggtgtagc-3	353
*Kuzbanian*	E33199	58	5-gcggtgcagattagtagatgc-3	5-aggcactaggaagaaccaagg-3	352
*Presenillin*	XM_007441	58	5-agactggaacacaaccatagcc-3	5-caatagtgcaaggtggtcagg-3	360
*Manic- Fringe*	U94352	60	5-gtggaatgatggcagaatcc-3	5-gctgtggatgattgtcttgg-3	384
*Radical- Fringe*	U94353	58	5-tgacaattatgtgaacgcaagg-3	5-ccaggtgagagtggaagagg-3	347
*Numb*	AF171938	58	5-cgaccgatggttagaagagg-3	5-gaatgtctgctgcctgacc-3	367
*Mastermind*	NM_014757	58	5-tcagaactccgcgaataacc-3	5-tgctggctgctgagatagg-3	351
*HES 1*	NM_005524	58	5-ccagtttgctttcctcattcc-3	5-tcttctctcccagtattcaagttcc-3	254
*Deltex*	NM_004416	61	5-gatgcctgtgaatggtctgg-3	5-ggatggtgatgcagatgtcc-3	362
*HERP 2*	AF232239	62	5-gaggtggagaaggagagtgc-3	5-ctccgatagtccatagcaagg-3	314
*TSL 1*	NM_005077	58	5-ccaaggtctgcttctcatgc-3	5-gtacttgtcaggcttgttcacg-3	320
*TSL 4*	XM_042357	58	5-caaggcagagctgacatcc-3	5-cacattgctgttctccatcc-3	346

**Table 2:** mRNA expression of elements of the Notch pathway

	**Receptors**		**Ligands**				**Modulators**					**Downstream effectors**				**Cleavage enzymes**	
**Case**	***Notch 2***	***Notch 3***	***Jagged 1***	***Jagged 2***	***Delta-like-1***	***Delta-like-2/4***	***Numb***	***M. Fringe***	***R. Fringe***	***Deltex***	***Mastemind***	***HES l***	***HERP 2***	***TSL 1***	***TSL 4***	***Kuzbanian***	***Presenillin***
*Ovarian adenocarcinoma (N=17)*																	
																	
1	+	+	+	+	+	+	+	+	+	0	+	+	+	+	+	+	+
2	+	+	+	+	+	+	+	+	+	+	+	+	0	+	+	+	+
3	0	+	+	0	0	+	+	+	0	+	0	+	0	+	+	0	+
4	+	+	+	+	+	+	+	+	+	+	0	+	0	0	+	+	+
7	+	+	+	+	+	+	+	+	+	0	0	+	0	+	+	+	+
10	+	+	+	+	+	+	+	+	+	+	+	+	0	+	+	+	+
11	+	0	+	+	+	+	+	+	0	+	0	+	0	+	0	+	+
13	+	+	+	+	+	+	+	+	+	+	+	+	0	+	+	+	+
16	+	+	+	+	+	+	+	+	+	+	+	+	0	+	+	+	+
17	+	+	+	+	+	+	+	+	+	+	+	+	0	+	+	+	+
19	0	+	+	+	+	+	+	0	0	+	+	+	0	+	+	+	+
20	+	+	+	+	+	+	+	+	+	+	+	+	0	+	+	+	+
21	+	+	+	+	+	+	+	+	+	+	+	+	+	+	+	+	+
23	+	0	+	0	+	+	+	+	+	+	0	+	0	+	+	+	+
26	+	+	+	+	+	+	+	+	+	+	+	+	0	+	+	+	+
31	+	+	+	+	+	+	+	0	+	+	+	+	0	+	+	+	+
37	+	+	+	+	+	+	+	0	+	0	+	+	0	+	+	+	+
																	
Frequencies	88%	88%	100%	88%	94%	100%	100%	82%	82%	82%	70%	100%	12%	94%	94%	94%	100%
	15/17	15/17	17/17	15/17	16/17	17/17	17/17	14/17	14/17	14/17	12/17	17/177	12/17	16/17	16/17	16/17	17/17
																	
*Borderline tumours (N=3)*																	
9	0	+	+	0	0	+	+	+	0	0	0	+	0	0	0	+	+
33	+	0	+	+	+	+	+	0	+	+	0	+	0	+	+	+	+
34	+	+	+	+	+	+	+	0	+	+	+	+	0	+	+	+	+
																	
Frequencies	67%	67%	100%	67%	67%	100%	100%	33%	67%	67%	33%	100%	0%	67%	67%	100%	100%
	2/3	2/3	3/3	2/3	2/3	3/3	3/3	1/3	2/3	2/3	1/3	3/3	0/3	2/3	2/3	3/3	3/3
																	
*Ovarian adenomas (N=12)*																	
14	+	0	+	+	0	+	+	0	+	+	0	+	0	+	+	+	+
15	+	+	+	+	0	+	+	0	+	+	+	+	0	0	+	+	+
24	+	+	+	0	+	+	+	0	+	+	+	+	0	0	+	+	+
25	+	+	+	0	0	+	+	0	+	+	+	+	0	0	+	+	+
27	+	+	+	0	0	+	+	0	0	+	+	+	0	0	0	+	+
28	0	+	+	0	0	+	0	0	+	+	0	+	0	0	+	+	+
29	+	+	+	0	0	+	+	0	+	+	+	+	0	0	+	+	+
32	+	+	+	+	+	+	+	+	+	+	+	+	0	+	+	+	+
35	+	+	+	+	0	+	+	0	+	+	+	+	0	+	+	+	+
36	+	+	+	+	+	+	+	0	+	+	+	+	0	+	+	+	+
38	0	+	+	0	+	+	0	+	0	+	0	+	0	+	+	0	+
39	+	+	+	+	+	+	+	0	+	+	+	+	0	+	+	+	+
																	
Frequencies	83%	92%	100%	50%	58%	100%	83%	17%	83%	100%	75%	100%	0%	50%	92%	92%	100%
	10/12	11/12	12/12	6/12	7/12	12/12	10/12	2/12	10/12	12/12	9/12	12/12	0/12	6/12	11/12	11/12	12/12
																	
*Cell lines (N=4)*																	
2780	0	+	+	+	0	0	+	+	+	+	+	+	0	+	+	+	+
2008	+	+	+	0	+	0	+	0	+	0	0	+	0	0	0	+	0
OVCAR-3	+	+	+	0	0	0	+	+	+	0	+	+	0	0	0	0	+
IOSE 144	0	0	+	0	0	0	0	+	0	0	+	+	0	0	+	0	+
																	
Frequencies	50%	25%	100%	25%	25%	0%	25%	75%	75%	25%	75%	100%	0%	25%	50%	50%	75%
	2/4	1/4	4/4	1/4	1/4	0/4	1/4	3/4	3/4	1/4	3/4	4/4	0/4	1/4	2/4	2/4	3/4

0=no signal detected; +=mRNA detected by RT–PCR.

The results of duplicate experiments were identical.

**Table 3:** Western blot analysis of Notch 1-EC (extracellular domain) and HESl protein

**Case**	**Notch l-EC**	**HES l**
*Ovarian adenocarcinomas*		
		
1	+	+
2	+	+
3	ND	ND
4	+	+
7	+	++
10	+	++
11	+	+
13	+	+
16	+	(+)
17	+	+
19	+	+
*20*	+	+
21	++	++
23	+	+
26	+	+
31	+	++
37	+	+
		
*Borderline tumours*		

9	+	++
33	+	+
34	+	++
		
*Ovarian adenomas*		

14	+	+
15	(+)	+
24	ND	ND
25	+	+
27	(+)	+
28	+	+
29	(+)	(+)
32	ND	ND
35	ND	ND
36	+	(+)
38	+	0
39	+	(+)
		
*Cell lines*		

A2780	+++	+++
2008	+	+++
OVCAR-3	++	+++
IOSE 144	+	+++

ND=not done; 0=negative; (+)=weak positive; +=positive; ++=marked positive; +++=strong positive.
